# Short stature in pre-pubertal children with X-linked hypophosphatemia

**DOI:** 10.1530/EC-24-0605

**Published:** 2025-05-31

**Authors:** Hanting Liang, Wenting Qi, Chenxi Jin, Cong Zhang, Yushuo Wu, Xiaosen Ma, Qianqian Pang, Ruizhi Jiajue, Yue Chi, Wei Liu, Yan Jiang, Ou Wang, Mei Li, Xiaoping Xing, Jiajun Zhao, Weibo Xia

**Affiliations:** ^1^ Department of Endocrinology, Key Laboratory of Endocrinology, National Commission of Health, State Key Laboratory of Complex Severe and Rare Diseases, Peking Union Medical College Hospital, Chinese Academy of Medical Sciences and Peking Union Medical College, Beijing, China; ^2^ Department of Endocrinology, The Second Affiliated Hospital, Zhejiang University School of Medicine, Hangzhou, Zhejiang, China; ^3^ Department of Endocrinology and Metabolism, Beijing Tsinghua Changgung Hospital, Tsinghua University, Beijing, China; ^4^ Department of Endocrinology, China-Japan Friendship Hospital, Beijing, China; ^5^ Department of Endocrinology, Shandong Provincial Hospital Affiliated to Shandong First Medical University; Shandong Provincial Key Laboratory of Endocrinology and Lipid Metabolism; Shandong Institute of Endocrine & Metabolic Disease, Jinan, Shandong, China

**Keywords:** X-linked hypophosphatemia, PHEX, height, short stature, rickets severity score

## Abstract

**Objective:**

Short stature is a characteristic of X-linked hypophosphatemia (XLH). We aim to explore the factors that influence the height of pre-pubertal children with XLH.

**Methods:**

Based on a randomized clinical trial of high/low doses of active vitamin D with neutral phosphate treatment for XLH children, we recruited 124 pre-pubertal children with XLH, and 46 participants completed the 24-month follow-up. Participants were separated into the short stature (height Z score < −2) and non-short stature groups (height Z score ≥ −2). Height, medication history, biochemical parameters, the Thacher Rickets Severity Score (RSS), and bone age were evaluated.

**Results:**

At baseline, 50.8% of participants were short stature. The height Z score of males (−2.35 ± 1.18) was significantly lower than that of females (−1.86 ± 1.03), *P* = 0.014. The height Z score had negative correlations with age when enrolled, initial age of medication, and RSS (β: −0.327∼−0.251, *P* < 0.01), but had a positive correlation with calcium-phosphorus product (β: 0.213, *P* = 0.015). Compared to the non-short stature group, the proportion of delayed bone age was higher in the short stature group (10.0 vs 42.9%, *P* < 0.001). At the 24-month follow-up, the median height Z score increased from −1.91 to −1.74 (*P* = 0.002), whose improvement had no significant differences between groups of male/female, high/low doses of calcitriol, and non-truncating/truncating variants.

**Conclusion:**

In pre-pubertal children with XLH, a higher height Z score has associations with females, early initiation of treatment, better bone mineralization, and milder rachitic lesions. Conventional therapy improves their heights, but the efficacy does not depend on sex, active vitamin D dosage, or variant type.

## Introduction

X-linked hypophosphatemia (XLH) is the most common type of hereditary hypophosphatemia rickets/osteomalacia, with an estimated prevalence of one in 20,000 and an incidence of 3.9–5 per 100,000 live births (1). Since phosphate-regulating endopeptidase X-linked (*PHEX*) gene was identified as the pathogenic gene of XLH in 1995 (2), 870 variants in the *PHEX* gene have been reported as of April 30, 2021 (3). Loss-of-function mutations of the *PHEX* gene lead to the increase of circulating fibroblast growth factor 23 (FGF23), resulting in hypophosphatemia by reducing resorption of phosphate in the renal proximal tubule and synthesis of 1,25-dihydroxyvitamin D (1,25-(OH)_2_D3) (4). The characteristic clinical manifestations of XLH typically manifest within the first 2 years of life, including progressive lower extremity bowing, bone deformities, growth impairment with disproportionate short stature, bone and joint pain, and recurrent dental abscesses (5). These issues aggravate the disease burden and decrease the quality of life in patients with XLH (5, 6).

Short stature is defined as a height below the third percentile or more than two standard deviations (SD) below the mean height for an age- and sex-matched reference population (7, 8). Among affected individuals with XLH, 55–70% of them manifest short stature, whose height Z score is less than −2 (6, 9), and the height has more impairment in adults than juveniles (9, 10). Both conventional therapy (neutral phosphate combined with active vitamin D) and novel antibody targeting FGF23 (Burosumab) can correct serum phosphate and improve active lesions of rickets to some extent, but the height improvement is limited (11, 12). With the amelioration of clinical manifestations and serum phosphate during treatment, patients with XLH and their guardians will pay more attention to the benefits of height improvement. Although XLH has high clinical heterogeneity and the height of affected individuals varies from person to person (13), several studies have indicated that height in XLH patients was associated with sexes, the level of serum phosphate, and early diagnosis and treatment (14, 15, 16). However, the associations between height and the level of insulin-like growth factor-1 (IGF-1), bone age (BA), and Thacher rickets severity score (RSS) remain unknown.

Based on a randomized clinical trial of high/low doses of active vitamin D with neutral phosphate treatment for XLH children, this study aims to ascertain the proportion of short stature in a group of Chinese pre-pubertal children with XLH and explore differences between those with and without short stature in aspects of clinical manifestations, biochemical characteristics, and radiological features. Moreover, we also analyzed the height change pre- and post-treatment among participants who completed 24 months of follow-up and investigated whether height change was different between participants with different sexes, active vitamin D doses, and variant types of the *PHEX* gene. By analyzing the height of patients with XLH and relevant influence factors, this study may provide inspiration and research focus in this field for endocrinologists and pediatricians.

## Subjects and methods

### Subjects

This study is a post hoc analysis from a single-center, randomized, open-label, prospective trial called ‘Using different doses of active vitamin D combined with neutral phosphate in children with XLH’ (NCT03820518) (11). Affected children aged 0–12 years old were recruited. Inclusion criteria: i) confirmed pathogenic *PHEX* gene variants in affected individuals; ii) pre-pubertal participants. Pre-pubertal status is defined as Tanner stage 1, including no glandular breast tissue palpable in females, testicular volume less than 4 mL or long axis less than 2.5 cm in males, and no pubic hair in both males and females (17); iii) willing to join this study with good compliance. Exclusion criteria: i) children in pubertal initiation, whose Tanner stage is 2 or above; ii) accepting recombinant human growth hormone therapy within 12 months before the first visit; iii) used or using drugs for delaying puberty, such as gonadotropin-releasing hormone antagonist; iv) used aluminum hydroxide, steroid, acetazolamide, or thiazide drugs within 7 days before the first visit; v) the level of parathyroid hormone (PTH) is 2.5 times over the upper limit of the normal range (>195 ng/mL); vi) planning to accept orthopedic surgery in the next 12 months; vii) poor compliance. All participants agreed to join this trial and their parents or guardians signed the informed consent. This study was approved by the Ethics Committee of Peking Union Medical College Hospital (JS-1824) and in accordance with the Declaration of Helsinki.

### Study design

The detailed study design has been described previously (11). In brief, participants were randomly assigned into two groups according to a computer-generated random numbers table. Participants in the high dose group and low dose group intake calcitriol with a dosage of 40 or 20 ng/kg per day, respectively. Participants in both groups intake neutral phosphate with a dosage of 30 mg/kg per day, divided into four to five times per day. They were followed up at baseline, 3, 6, 12, 18, and 24 months. In this study, we analyzed baseline characteristics in all participants, whereas the height change pre- and post-treatment was analyzed in those who completed the 24-month follow-up.

### Clinical information collection

Information about family history, previous medication, previous orthopedic treatment, and clinical manifestations was collected from every participant at baseline. Previous medication referred to conventional treatment with calcitriol and neutral phosphate, and the number of participants receiving previous medication and the course of treatment were recorded. Previous orthopedic treatment mainly referred to orthopedic surgery or orthoses of the lower extremities. Lower limb deformities mainly included genu varum and genu valgum. Inter-femoral, inter-knee, and inter-tibial distances in patients with genu varum, and inter-femoral, inter-tibial, and inter-malleolar distances in patients with genu valgum were measured. We selected the maximum distance among these data to evaluate the severity of the lower extremities. We measured the height and weight of all participants at every visit. Z scores of height, weight, and BMI were calculated according to the standardized growth charts for Chinese children and adolescents (18, 19). Based on the height Z score at baseline, all participants were separated into the short stature (height Z score < −2) and non-short stature groups (height Z score ≥ −2).

### Biochemical measurements

The fasting blood sample of each participant was collected in the morning, approximately one hour after taking phosphate. The fresh serum was used to measure the levels of total calcium, phosphate, alkaline phosphatase (ALP), creatinine (Cr), 24 h urine calcium (24 h UCa), and 24 h urine phosphate (24 h UP) by an auto-analyzer (Beckman Coulter AU5800, USA). Total 25-hydroxyvitamin D (T25OHD) was measured by the electrochemiluminescence immunoassay method (Roche Cobas E601 analyzer, Roche Diagnostics, Switzerland). The level of 1,25-(OH)_2_D3 was measured by enzyme-linked immunosorbent assay (DIASORIN, USA). Levels of parathyroid hormone (PTH) and IGF-1 were measured by Immulite 2000 (Siemens, Germany). Reference ranges of all these parameters were obtained from the Department of Laboratory Medicine, PUMCH. Z scores of serum phosphate, ALP, and IGF-1 were calculated according to the reference ranges provided by the Department of Laboratory Medicine, PUMCH.

### Radiological assessment

All participants underwent X-ray examinations of both hands and knees at baseline. Two experienced radiologists assessed the RSS on bilateral wrists and knees (20) and evaluated BA on the left hand according to the Greulich-Pyle atlas (21). When BA was over 1 year less than chronological age (CA), the condition was defined as delayed BA. The absolute value of ‘BA-CA’ no more than 1 year was considered as ‘BA equal to CA’.

### Verification of *PHEX* gene variants

The genomic DNA of every participant was extracted from peripheral blood for further genetic tests (QIAamp DNA Micro; QIAGEN, Germany). Sanger sequencing and multiplex ligation-dependent probe amplification (MLPA) analysis were performed to detect pathogenic variants in the *PHEX* gene as previously described (10). According to the type of variants, missense mutations and non-frameshift insertion or deletion were classified as ‘non-truncating variants’, whereas ‘truncating variants’ included nonsense mutations, frameshift insertion or deletion, and splicing mutations.

### Statistical analysis

Statistical analysis was conducted using SPSS version 24.0. The Shapiro–Wilk test was used to determine the normality of all continuous variables. Normally distributed variables were depicted as mean ± standard deviation, whereas abnormally distributed variables were described as median (interquartile range, IQR). Normally distributed variables and abnormally distributed variables were compared by Student’s *t*-test and Mann–Whitney U test between two groups, respectively. Classified variables between two groups were compared by the chi-square test or Fisher’s exact test. Bivariate analysis was performed to detect correlations between the IGF-1 Z score and other biochemical parameters by Pearson analysis or Spearman analysis. Univariable and multivariable linear regression analyses were conducted to evaluate associations between height Z score and general, biochemical, and radiological parameters at baseline. Height change pre- and post-treatment (at baseline vs 24 months) in all participants or subgroups was compared by Wilcoxon signed-rank test or paired *t*-test. Height change pre- and post-treatment between different subgroups was compared by one-way ANCOVA. *P* value <0.05 was considered a statistically significant difference.

## Results

### General characteristics of the whole cohort at baseline

A total of 124 participants were included at baseline, among which 50.8% were short stature. Table 1 summarizes their general characteristics and clinical manifestations. Z scores of height and weight in the short stature group were significantly lower than those in the non-short stature group, which were −2.89 (0.77) vs −1.36 (0.85) for height Z score, and −1.09 (1.16) vs −0.09 (1.35) for weight Z score (both *P* values < 0.001). The initial age of medication was significantly earlier in the non-short stature group compared to the short stature group (1.73 (1.42) years old vs 2.50 (2.20) years old, *P* = 0.001). The age at enrollment, sex ratio, BMI and its Z score, family history of XLH, percentages of previous medication with calcitriol and neutral phosphate, previous orthopedic treatment, and proportions of typical clinical manifestations were comparable between the two groups.

**Table 1:** General characteristics of patients with XLH between non-short stature and short stature groups at baseline.

	Non-short stature (*n* = 61)	Short stature (*n* = 63)	*P* value
Age when enrolled (years old)	3.50 (3.91)	4.50 (3.60)	0.050
Female	62.3% (38/61)	46.0% (29/63)	0.075
Height (cm)	97.5 (26.5)	94.5 (23.5)	0.601
Height Z score	−1.36 (0.85)	−2.89 (0.77)	**<0.001**
Weight (kg)	15.5 (9.5)	15.0 (8.0)	0.301
Weight Z score	−0.09 (1.35)	−1.09 (1.16)	**<0.001**
BMI (kg/m^2^)	16.89 (2.30)	16.83 (2.05)	0.519
BMI Z score	0.94 (1.67)	1.06 (1.08)	0.456
Family history	44.3% (27/61)	49.2% (30/61)	0.717
Treatment before enrolled			
Medical history of calcitriol and neutral phosphate	78.7% (48/61)	82.5% (52/63)	0.653
Initial age of medication (years old)	1.73 (1.42)	2.50 (2.20)	**0.001**
Duration of previous oral medication (months)	28.5 (35.3)	20.0 (31.0)	0.593
Previous orthopedic treatment	6.6% (4/61)	12.7% (8/63)	0.364
Symptoms and signs			
Weakness	43.3% (26/60)	59.0% (36/61)	0.103
Waddling gait	79.7% (47/59)	85.2% (52/61)	0.477
Bone pain	47.5% (29/61)	49.2% (30/61)	>0.999
VAS	2.0 (0.0)	2.0 (2.0)	0.219
Dental abscess			0.096
Negative	66.1% (39/59)	50.0% (31/62)	
Occasional	20.3% (12/59)	21.0% (13/62)	
Frequent	13.6% (8/59)	29.0% (18/62)	
Cranial deformities	66.7% (38/57)	75.8% (47/62)	0.313
Thoracic deformities	93.4% (57/61)	93.7% (59/63)	>0.999
Bracelet and anklet sign	80.0% (48/60)	88.7% (55/62)	0.218
Lower extremity deformities	88.5% (54/61)	95.2% (60/63)	0.202
The maximum distance of deformities in lower extremities (cm)	5.0 (2.5)	5.3 (3.0)	0.353
Truncating variants	75.4% (46/61)	87.3% (55/63)	0.108

Non-short stature: height Z score ≥ −2.0; short stature: height Z score < −2.0. Previous orthopedic treatment includes surgery and orthoses. Lower extremity deformities include genu varum and genu valgum. Truncating variants: nonsense mutations, frameshift insertion or deletion, and splicing mutations; non-truncating variants: missense mutations and non-frameshift insertion or deletion. Bold indicates statistical significance. Abbreviations: XLH, X-linked hypophosphatemia; VAS, visual analog score.

### Biochemical parameters at baseline between the two groups and bivariate analysis

All biochemical parameters at baseline are listed in Table 2. Levels of serum calcium, serum phosphate, and its Z score were significantly inferior to those of the non-short stature group (all *P* values <0.01). The proportion of those whose calcium-phosphorus product was less than 30 mg^2^/dL^2^ reached 84.7%. Calcium-phosphorus product was significantly lower in the short stature group compared to the non-short stature group (23.80 (4.89) mg^2^/dL^2^ vs 25.86 (4.78) mg^2^/dL^2^, *P* = 0.026 after adjustment for age). Compared to the non-short stature group, the level of IGF-1 and its Z score were significantly lower in the short stature group (all *P* values <0.05). Other biochemical parameters showed no significant differences between the two groups. We further investigated the correlation between the IGF-1 Z score and other biochemical parameters. However, there were no significant correlations between the IGF-1 Z score and levels of calcium, phosphate, ALP, Cr, PTH, T25OHD, and 1,25-(OH)_2_D3 (Table 3).

**Table 2:** Biochemical parameters and radiological characteristics of patients with XLH between non-short stature and short stature groups at baseline.

	Non-short stature (*n* = 61)	Short stature (*n* = 63)	*P* value
Ca (mmol/L)	2.43 ± 0.09	2.38 ± 0.08	**0.002**
P (mmol/L)	0.84 (0.12)	0.79 (0.18)	**0.005**
Z score of P	−3.85 (0.94)	−4.25 (0.85)	**0.003**
Ca x P (mg^2^/dL^2^)	25.86 (4.78)	23.80 (4.89)	**0.001^†^**
ALP (U/L)	580.2 ± 167.9	610.5 ± 147.1	0.287
Z score of ALP	4.15 ± 2.41	4.47 ± 2.06	0.538
Cr (μmol/L)	27.0 (10.8)	27.0 (7.3)	0.285
PTH (pg/mL)	45.3 (30.0)	58.6 (52.0)	0.076
T25OHD (ng/mL)	29.6 (18.5)	26.3 (12.9)	0.111
1,25-(OH)_2_D3 (pg/mL)	38.1 (38.1)	52.7 (34.7)	0.068
24 h UCa (mmol/24 h)	0.46 (1.07)	0.51 (0.91)	0.778
24 h UP (mmol/24 h)	24.9 (20.6)	19.0 (18.4)	0.168
IGF-1 (ng/mL)*	131.0 (84.0)	99.5 (59.0)	**0.034**
Z score of IGF-1*	0.11 ± 0.57	−0.30 ± 0.92	**0.042**
RSS	5.0 (2.0)	6.0 (2.0)	**<0.001**
Delayed bone age	10.0% (5/50)	42.9% (21/49)	**<0.001**
BA-CA (years)	−0.10 (0.50)	−0.75 (1.28)	**<0.001**

Non-short stature: height Z score ≥ −2.0; short stature: height Z score < −2.0. Bold indicates statistical significance.

* IGF-1 and its Z score are available in 59 participants with XLH at baseline.

^†^ *P* = 0.026 with adjustment for age.

Abbreviations: XLH, X-linked hypophosphatemia; Ca, calcium; P, phosphate; Ca x P, calcium-phosphorus product; ALP, alkaline phosphatase; Cr, creatinine; PTH, parathyroid hormone; T25OHD, total 25-hydroxyvitamin D; 1,25-(OH)_2_D3, 1,25-dihydroxyvitamin D; 24 h UCa, 24-h urinary calcium; 24 h UP, 24-h urinary phosphate; IGF-1, insulin-like growth factor 1; RSS, rickets severity score; BA, bone age; CA, chronological age. Reference range for each parameter from Peking Union Medical College Hospital: Ca, 2.13–2.7 mmol/L; P, 0–1 year old: 1.55–2.65 mmol/L, 1–4 years old: 1.25–2.10 mmol/L, 4–12 years old: 1.20–1.80 mmol/L, 12–16 years old: 0.95–1.75 mmol/L, 16–19 years old: 0.90–1.45 mmol/L, over 19 years old: 0.81–1.45 mmol/L; ALP, male: 1–10 years old: 145–420 U/L, 10–12 years old: 130–560 U/L, 12–14 years old: 200–495 U/L, 14–16 years old: 130–525 U/L, 16–19 years old: 65–260 U/L, over 19 years old: 30–120 U/L; female: 1–10 years old: 145–420 U/L, 10–12 years old: 130–560 U/L, 12–14 years old: 105–420 U/L, 14–16 years old: 70–230 U/L, 16–19 years old: 50–130 U/L, over 19 years old: 27–107 U/L; Cr, 18–69 μmol/L; PTH, 12–68 pg/mL; T25OHD, ≥30 ng/mL; 1,25-(OH)_2_D3, 19.6–54.3 pg/mL.

**Table 3:** Correlation analysis between IGF-1 Z score and other biochemical parameters in patients with XLH.

	Correlation coefficient	*P* value
Ca (mmol/L)*	0.058	0.660
P (mmol/L)	0.189	0.152
Z score of P	0.096	0.467
P (LLN)	0.176	0.182
Ca x P (mg^2^/dL^2^)	0.206	0.117
ALP (U/L)*	0.060	0.654
Z score of ALP*	0.036	0.785
ALP (ULN)*	0.045	0.737
Cr (μmol/L)	0.054	0.683
PTH (pg/mL)	0.087	0.516
T25OHD (ng/mL)	−0.088	0.555
1,25-(OH)_2_D3 (pg/mL)	−0.068	0.666

* Pearson correlation analysis. Other parameters: Spearman correlation analysis.

Abbreviations: XLH, X-linked hypophosphatemia; Ca, calcium; P, phosphate; ALP, alkaline phosphatase; Cr, creatinine; PTH, parathyroid hormone; T25OHD, total 25-hydroxyvitamin D; 1,25-(OH)_2_D3, 1,25-dihydroxyvitamin D; IGF-1, insulin-like growth factor 1; Ca x P, calcium-phosphorus product.

### Comparison of RSS and BA between the short stature and non-short stature groups at baseline

As Table 2 shows, RSS was significantly higher in the short stature group than in the non-short stature group (6.0 (2.0) vs 5.0 (2.0), *P* < 0.001). The percentage of delayed BA in patients with short stature was significantly higher than in those without short stature, accounting for 42.9 and 10.0%, respectively (*P* < 0.001). The differences between BA and CA were −0.75 (1.28) years in the short stature group and −0.10 (0.50) years in the non-short stature group, respectively (*P* < 0.001). In addition, bivariate analysis showed that RSS had a negative correlation with the difference between BA and CA for the whole cohort at baseline (Spearman analysis, rs = −0.211, *P* = 0.041).

### Influence factors on height Z score at baseline

**Figure 1:**
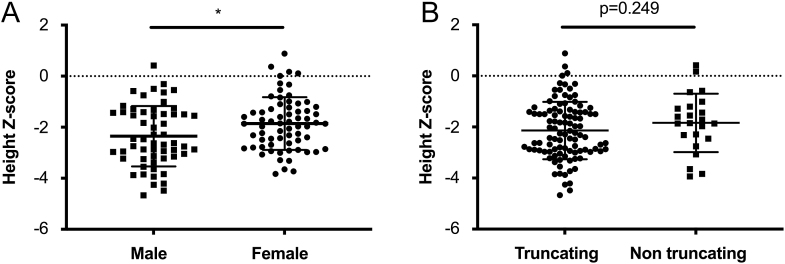
Comparison of height Z score at baseline between different subgroups. (A) Height Z score between males and females. (B) Height Z score between XLH patients with truncating and non-truncating variants of the *PHEX* gene. Abbreviations: XLH, X-linked hypophosphatemia; PHEX, phosphate-regulating endopeptidase homolog, X-linked. *P < 0.05.

In the whole cohort, the height Z score of males was significantly inferior to that of females at baseline (males vs females: −2.35 ± 1.18 vs −1.86 ± 1.03, *P* = 0.014, Fig. 1A). However, the height Z score at baseline had no significant difference between the truncating variants group (−2.14 ± 1.12) and the non-truncating variants group (−1.84 ± 1.14), *P* = 0.249 (Fig. 1B). As Table 4 shows, the height Z score at baseline had significantly negative correlations with age when enrolled (*β* = −0.251, *P* = 0.005) and initial age of medication (*β* = −0.361, *P* < 0.001) among all participants. Height Z score had a significantly positive association with the duration of previous oral medication with adjustment for age (*β* = 0.385, *P* = 0.004). As for biochemical parameters, height Z score was positively correlated to levels of calcium, phosphate and its Z score, calcium-phosphorus product, T25OHD, and Z score of IGF-1 (β: 0.187–0.370, all *P* values <0.05, Table 4). After adjustment for age, height Z score was still positively associated with calcium-phosphorus product (*β* = 0.213, *P* = 0.015). Height Z score at baseline had a negative correlation with RSS (*β* = −0.327, *P* < 0.001), whereas it was positively correlated with the difference between BA and CA (*β* = 0.422, *P* < 0.001) with adjustment for age.

**Table 4:** Correlations between height Z score and general, biochemical, and radiological parameters at baseline.

	*β*	*p*	*β*1	*p1*
Age when enrolled (years old)	−0.251	**0.005**	/	/
Initial age of medication (years old)	−0.361	**<0.001**	−0.321	**0.002**
Duration of previous oral medication (months)	0.054	0.591	0.385	**0.004**
VAS	−0.259	0.059	−0.230	0.104
Ca (mmol/L)	0.348	**<0.001**	0.328	**<0.001**
P (mmol/L)	0.187	**0.037**	0.163	0.063
Z score of P	0.203	**0.024**	/	/
Ca x P (mg^2^/dL^2^)	0.239	**0.008**	0.213	**0.015**
ALP (U/L)	−0.060	0.510	−0.086	0.333
Z score of ALP	−0.018	0.847	/	/
PTH (pg/mL)	−0.161	0.078	−0.157	0.078
T25OHD (ng/mL)	0.312	**0.002**	0.225	0.051
1,25-(OH)_2_D3 (pg/mL)	−0.121	0.301	−0.155	0.168
Z score of IGF-1	0.370	**0.004**	/	/
RSS	−0.361	**<0.001**	−0.327	**<0.001**
BA-CA (years)	0.453	**<0.001**	0.422	**<0.001**

*p*: without adjustment; *p1*: with adjustment for age when enrolled. Bold indicates statistical significance. Abbreviations: VAS, visual analog score; Ca, calcium; P, phosphate; LLN, lower limit of the normal range; ALP, alkaline phosphatase; ULN, upper limit of the normal range; Cr, creatinine; PTH, parathyroid hormone; T25OHD, total 25-hydroxyvitamin D; 1,25-(OH)_2_D3, 1,25-dihydroxyvitamin D; IGF-1, insulin-like growth factor 1; Ca x P, calcium-phosphorus product; RSS, rickets severity score; BA, bone age; CA, chronological age.

### Height change after treatment in the whole cohort or between different subgroups

**Figure 2:**
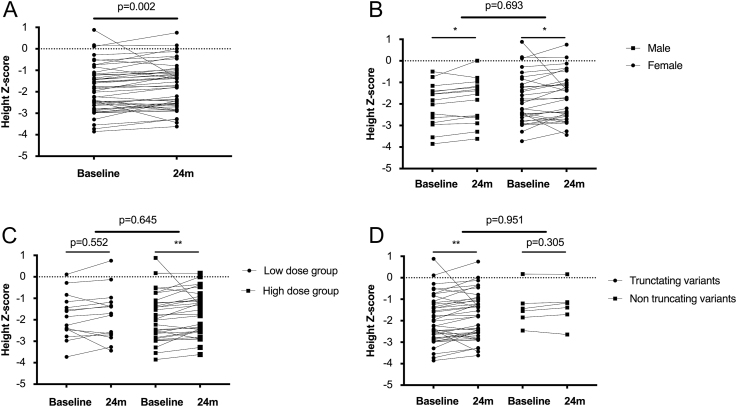
Change of height Z score from baseline to the last visit. (A) Z score of height change among 46 participants. (B) Z score of height change between males and females. (C) Z score of height change between the low-dose group and the high-dose group. (D) Z score of height change between the truncating variants group and the non-truncating variants group. Truncating variants: nonsense mutations, frameshift insertion or deletion, and splicing mutations; non-truncating variants: missense mutations and non-frameshift insertion or deletion. *P < 0.05; **P < 0.01.

Forty-six participants completed the 24-month follow-up. With regular treatment of calcitriol and neutral phosphate, the median height Z score increased from −1.91 (−2.67, −1.19) at baseline to −1.74 (−2.59, −1.07) at the 24th month in the whole cohort, *P* = 0.002 (Fig. 2A). Height Z score significantly improved after treatment both in males (−2.03 ± 0.99 vs −1.85 ± 1.02, *P* = 0.015) and females (−1.80 ± 1.12 vs −1.70 ± 1.08, *P* = 0.032), but the improvement of height Z score had no significant difference between males and females (*P* = 0.693, Fig. 2B). Height Z score had no significant change in the low dose group (−1.84 ± 1.03 vs −1.77 ± 1.16, *P* = 0.552), while it significantly improved from −1.89 ± 1.11 at baseline to −1.74 ± 1.01 at the last visit in the high dose group (*P* = 0.002). But the change of height Z score was comparable between the two groups with different calcitriol doses (*P* = 0.645, Fig. 2C). Individuals with truncating variants had a significant improvement in height Z score (−1.95 ± 1.09 vs −1.82 ± 1.07, *P* = 0.005), whereas the height Z score had no significant change in those with non-truncating variants (−1.39 ± 0.88 vs −1.32 ± 0.91, *P* = 0.305). Similarly, the improvement of height Z score had no significant difference between the two groups with truncating and non-truncating variants (*P* = 0.951, Fig. 2D).

## Discussion

In this study, based on the cohort of XLH accepting high/low doses of active vitamin D with neutral phosphate treatment, we explore the influence factors on height among pre-pubertal children with XLH and find that short stature in pre-pubertal children with XLH is associated with sex, initial age of medication, and duration of previous oral medication. This study also innovatively demonstrates that lower levels of calcium-phosphorus product and IGF-1 Z score, higher RSS, and delayed BA have correlations with short stature among these affected children.

Regarding clinical manifestations, disproportionate short stature and deformities in lower extremities are predominant characteristics in XLH patients (6, 22, 23). Approximately 50% of affected individuals manifest growth retardation (6, 23), and the mean Z score of adult height of XLH patients is impaired to −1.9 (24). According to our previous study, height impairment is more severe in China, with a mean height Z score of −2.4 in juveniles and −3.5 in adults, respectively (10). When patients start learning to stand or toddle, bearing weight causes lower extremity bowing, resulting in height impairment, and this phenomenon aggravates with increasing age (15). However, the proportion and severity of lower extremity deformities have no significant differences between the non-short stature and short stature groups in this cohort, indicating that bowing leg is not the only explanation for short stature in XLH patients (22).

As for biochemical parameters, calcium-phosphorus product, Z scores of IGF-1 and phosphate have significantly positive associations with height Z score among children with XLH. Calcium-phosphorus product has seldom been applied for evaluating the disorder condition of pre-pubertal children with XLH. Normalization of calcium-phosphorus product is meaningful to bone mineralization, while this parameter of less than 30 mg^2^/dL^2^ will result in insufficient bone mineralization (25, 26). At baseline, according to calcium-phosphorus product below 30 mg^2^/dL^2^, we infer that approximately 85% of affected individuals have the problem of insufficient bone mineralization, and the level of calcium-phosphorus product is lower in the short stature group, indicating more severe impairment in bone mineralization. So maintaining a normal level of calcium-phosphorus product may improve long bone growth in XLH children. In this study, a low Z score of IGF-1 is significantly associated with short stature. Early research demonstrates that short stature in XLH patients is not associated with growth hormone/IGF-1 secretion defect (27), but cases of XLH combined with growth hormone deficiency have been reported later (28). IGF-1 interacts with phosphorus, and IGF-1 promotes positive phosphorus balance via increasing phosphorus resorption in the renal tubule (29). However, in the animal study, researchers found that serum IGF-1 levels in hypophosphatemic (Hyp) mice have no significant difference compared to those in normal mice (30). A previous study illustrates that a high level of ALP is related to the disease activity and severity of XLH (31). However, our study did not identify a significant correlation between the ALP level and height Z score among XLH children. While ALP predominantly reflects the activity of bone formation in the short term, prolonged mineralization insufficiency remains the primary contributor to impaired linear growth in this context.

The RSS is a crucial radiological indicator for evaluating the disease severity in XLH patients. A previous study shows a higher RSS is related to more severe height impairment (32). Similarly, RSS is higher in the short stature group than in the non-short stature group in this study. We suppose that this phenomenon may be associated with poor bone mineralization. The low level of calcium-phosphorus product in a child with XLH indicates poor bone mineralization in the metaphysis of long bones, mainly manifesting as short stature clinically and high RSS radiologically. Following Burosumab treatment, there is an improvement in height Z score and a decrease in RSS among children with XLH, which provides additional evidence of the close relationship between height and RSS (33, 34). The proportion of delayed BA is higher in the short stature group than in the non-short stature group. Whether delayed BA can be the explanation for short stature in XLH children to some extent? Indeed, the Z score of IGF-1 in the short stature group is inferior to that in the non-short stature group, with a mean value of −0.3 and not less than −2.0, which will not lead to the phenomenon of extremely delayed BA than CA. The delayed BA may be attributed to poor bone mineralization of the epiphysis. Affected individuals with delayed BA always have more physiological time for catch-up growth, but whether XLH children can get catch-up growth in the setting of bone mineralization defects remains unknown. A long-term study is needed to explore the association between BA and final adult height among XLH patients. There are close associations among height, RSS, and BA. So we think that both a high RSS and delayed BA are characteristics of poor bone mineralization in short stature children with XLH.

Among affected individuals with XLH, whether sex has an influence on the Z score of height varies from different study populations. Previous studies show that height Z score is comparable between males and females in adults (more than 18 years) and all ages of XLH patients (5 months to 58 years) (10, 24). However, there is less height loss in females than in males among pre-pubertal children with XLH in this study, which may result from the dosage effect of the *PHEX* gene on the skeletal phenotype (35). In addition, the same as this study, other researchers demonstrate no correlation between the genetic variant type and height among patients with XLH (10, 36). Regarding the pathogenic mechanism, the influence of the *PHEX* gene variant on height is not only related to abnormal bone mineralization caused by hypophosphatemia (22, 37, 38), but also associated with the effect of PHEX protein on growth plates (13). In wild-type mice, Phex is highly expressed in chondrocytes, osteoblasts, and osteocytes (39, 40). However, Hyp mice lack Phex protein, leading to cellular morphology alteration, loss of polarity, abnormal proliferation, decreased apoptosis, and impaired differentiation in hypertrophic chondrocytes (13, 37, 39, 41). In addition, there are changes of local regulators and growth factors in the growth plate of Hyp mice (13).

A previous study shows that XLH children who accept conventional treatment before 1 year old gain more height improvement (16). In this study, the initiation age of therapy has a negative correlation with height Z score. The growth curve for children with XLH shows decreased height gain by 1 year old (42), so early initiation of treatment is beneficial to height improvement among children with XLH. Height improvement does not depend on sex, variant type, and dosage of calcitriol (16). With 2 years of treatment of calcitriol and neutral phosphate, the mean value of height Z score increases significantly by 0.17 in pre-pubertal children with XLH, but their heights are still under the average level of the same sex and age children. Several studies explored the efficacy of recombinant human growth hormone (rhGH) on height in XLH children and showed that rhGH was beneficial for height improvement in affected children with XLH (43, 44, 45, 46), especially in those combined with growth hormone deficiency and pre-pubertal children (28, 44), but the aggravated bone deformities and abnormal body disproportion should be concerned (13, 46). Burosumab, a human monoclonal antibody against FGF23, can improve linear growth among XLH children to some extent as a novel therapy (12, 33). However, the impact of long-term treatment of Burosumab on linear growth and final adult height of affected individuals with XLH remains unknown and needs further exploration (13).

The strength of this study is that we comprehensively analyzed the relevant factors of height among pre-pubertal children with XLH based on a longitudinal study, including clinical, biochemical, radiological, genetic, and therapeutic aspects. There are also limitations in this study. First, this study only enrolled pre-pubertal participants, so we cannot investigate the influence of puberty on height in patients with XLH. Second, we did not measure other anthropometric parameters for analysis, such as sitting height, arm span, and upper segment/lower segment ratio of the body. Third, approximately 50% (59/124) of participants had IGF-1 data at baseline, which might bring bias to the related result. Fourth, the number of participants who completed the 2-year follow-up was limited, only accounting for 37% (46/124). Finally, bone mineralization defects in XLH patients may affect the accuracy of BA evaluation and bring bias.

In conclusion, female sex, early initiation of treatment, a higher level of IGF-1, better bone mineralization, and milder rachitic lesions may be the beneficial factors for height among pre-pubertal XLH patients, but the height Z score is not correlated with variant types and recent XLH activity reflected by the ALP level. Conventional therapy with neutral phosphate and active vitamin D improves height in pre-pubertal children with XLH, but the efficacy on height improvement has no associations with sex, the dose of active vitamin D, and the variant types of the *PHEX* gene.

## Declaration of interest

The authors declare that there is no conflict of interest that could be perceived as prejudicing the impartiality of the work reported.

## Funding

This study was supported by the National Natural Science Foundation of China (82270938, 81970757, 81900811), the Chinese National Key Technology R & D Program, Ministry of Science and Technology (2021YFC2501700), the Chinese Academy of Medical Sciences-CAMS Innovation Fund for Medical Sciences (CIFMS-2021-I2M-1-002), and National high-level hospital clinical research funding (2022-PUMCH-D-006).

## Author contribution statement

WB Xia designed the study and revised the manuscript. HT Liang, WT Qi, CX Jin, C Zhang, YS Wu, XS Ma, and QQ Pang collected the blood samples and analyzed the genetic results. HT Liang, WT Qi, CX Jin, C Zhang, YS Wu, RZ Jiajue, Y Chi, W Liu, Y Jiang, O Wang, M Li, XP Xing, JJ Zhao, and WB Xia collected clinical information of patients. HT Liang did the statistical analysis and drafted the manuscript. All authors read and approved the final manuscript.

## Data availability

The raw datasets generated and/or analyzed during this study are not publicly available but are available from the corresponding author on reasonable request.

## Ethics approval

The study was performed with the approval of the Ethics Committee of Peking Union Medical College Hospital.

## Consent to participate

All of the subjects agreed to participate in this study and signed informed consent forms.

## Consent for publication

All authors agreed to publish the manuscript.
